# Pregnant women perceptions regarding their husbands and in-laws’ support during pregnancy: a qualitative study

**DOI:** 10.11604/pamj.2021.39.229.25659

**Published:** 2021-08-09

**Authors:** Sehrish Naz, Dildar Muhammad, Ashfaq Ahmad, Shabnam, Parveen Ali

**Affiliations:** 1 Institute of Nursing Sciences, Khyber Medical University, Peshawar, Pakistan,; 2 Himalaya College of Nursing, Mardan, Pakistan,; 3 The School of Nursing and Midwifery, University of Sheffield, United Kingdom

**Keywords:** Support, husbands, pregnancy, pregnant women, perceptions, in-laws

## Abstract

**Introduction:**

pregnancy is a stressful condition during which women require family and in-laws´ support. This study was aimed to explore the women´s perceptions regarding their husband and in-law´s support during pregnancy.

**Methods:**

by using qualitative exploratory design ten pregnant women in third trimester of pregnancy and living in joint family system were recruited through purposive sampling technique from a village of district Nowshehra, Khyber Pakhtunkhwa, Pakistan. Approval for conducting this study was obtained from Ethics Review Committee of Khyber Medical University. Data were collected from the recruited participants through face to face in-depth interviews. Data were analyzed through thematic analysis. One hundred open codes were generated from the data. Through axial coding, extra and unnecessary codes were omitted and then eleven categories were identified from open codes.

**Results:**

the identified categories were kept under three salient themes of lack of comprehensive support mechanism, physical and mental strain, and barriers to antenatal services. Perceived support of husbands and in-laws, needs and barriers to maternal and child health were discussed by the participants.

**Conclusion:**

the study findings suggest that family relationship quality might not be improved by taking interventions i.e. making policies only but the incorporation of health professionals´ support with family member´s behavior can improve maternal health.

## Introduction

Pregnancy is not a disease but a psychologically challenging period where a woman passes through several social, physical and psychological challenges in life [1]. During pregnancy, women need significant support from health care services, however, in patriarchal societies such as Pakistan, a lot of these decisions related to access to health services etc. are in the hands of husband and in-laws [2]. Women have limited autonomy and power of expression due to deeply rooted societal norms may lead pregnant women to depression and affect their pregnancy and fetal weight [3-7]. Worldwide, 10% of pregnant women experience stress and depression due to the autocratic style of their in-laws [8]. In addition, family values and beliefs, religion, level of education or awareness of the family members affect the psychological, physical and social wellbeing of pregnant women [5].

Mostly traditional families with rigid belief system think that medical care is not necessary in pregnancy and they don´t allow pregnant women to seek medical care due to which health seeking gets delay [9]. Delay in health seeking leads to undesirable health outcomes such as high fertility, undesirable and unwanted pregnancies, and medical complications in women [10]. During antenatal period, husbands and family members´ support is necessary to ensure healthy pregnancy outcomes [11]. Specially, husband´s presence at the time of delivery is fruitful because partner´s support strengthens and helps in reducing fear and anxiety during delivery [7].

A study reported that witnessing labor pain can help in family planning in future [8]. Social support in terms of emotional, cognitive guidance, positive feedback, and social reinforcement to pregnant women from their family members is associated with better mental health, buffering of risks and promotion of well-being [12]. Psychological support in terms of family members´ behavior and communication is another important factor which affects maternal mental health and boost up pregnancy outcomes. Lack of psychological support can lead to pregnancy complications i.e. deprived neurodevelopment of fetus, low birth weight of fetus, an increase in the rates of caesarean birth, prolonged and preterm labor which indicates that poor psychological support during pregnancy is strongly related to pregnancy complications [13]. Quality family relationship plays an important role in the pregnant women´s mental health and physical well-being [14]. There is strong disparity Pakistani cities as well as villages and gender inequalities including literacy, nutrition, employment and health care and determinants affect women´s health seeking behavior during pregnancy [15]. The current study was aimed at exploring the perceptions of pregnant women regarding their husbands and in-laws´ support during pregnancy.

## Methods

A qualitative exploratory study design was used to explore the perceptions of pregnant women regarding their husbands and in-laws´ support during pregnancy. Using purposive sampling technique, pregnant women who were in third trimester of pregnancy, house wives, living in joint family in the village of district Nowshehra, Pakistan were selected. Study was completed in six months. Sample size was decided upon the data saturation to produce sufficient in-depth information that can highlight the blueprints, types and aspects of the phenomenon of interest.

Permission was taken from the University´s ethical review board before the commencement of data collection. Rapport was built with the participants and research purpose and author´s information was fully disclosed then consent was secured for face to face individual in-depth interview as well as for audio recording. Language convenient to the participants was used during the interview. Semi-structured interviews were conducted individually. Interviews were audio recorded and field notes were taken.

Data was found saturated on tenth participant. Audio recorded information was then translated into English language and transcribed verbatim anonymously following each interview. Thematic analysis approach was used to analyze the data and analysis of the data was proceeding step by step [16].

Audio recorded information was listened one time and then data read and re-read and initial analytic induction was noted. Next, semantic and conceptual reading was done; all the data were coded, and the relevant data were extracted from the codes. Next, searching for the repetition or similarity in the initial codes was done. Next, themes were discovered from the coded data. The codes were then arranged relevant to each theme. Lastly, themes were reviewed and names were identified for each theme concisely and all the themes wrote in details which provided the readers a holistic view of the research.

## Results

A hundred (100) open codes were found out from the data. In the process of axial coding extra and unnecessary codes were omitted and then 11 categories were identified from open codes. The identified categories were kept under three major themes of lack of comprehensive support mechanism, physical and mental strain, barriers to antenatal services. Perceived support of husbands and in-laws and needs and barriers to maternal and child health were discussed by the participants.

**Lack of a comprehensive support mechanism:** overall, participants disclosed a lack of a comprehensive support mechanism in terms of emotional support, physical support, psychological support, housekeeping support, and financial support during pregnancy from their husband and in-laws. The participants in this study lived in joint families with complex family dynamics and power hierarchies outside of their control. The behaviours of husbands and in-laws were reported to be stress causing pregnant ladies which was affecting their health during pregnancy. Interviewees explained that their husbands showed careless behaviour towards them. In joint family system, their husbands, as reported by these women, wouldn´t say on behalf of them when their in-laws scolded them and where the husband´s support was direly need in household chores during pregnancy. They felt helpless when no one listened to their health problems.

Overall, these experiences were reported to be painful for these ladies as they felt helpless when they needed support the most. Participants complained of their husbands´ irresponsible behaviours like not doing any work or having education but not actively searching for any job, as these housewives were financially dependent on their husbands, this would leave their wives and children to suffer at home regarding their healthcare, these women mentioned, for example, as:

*“My husband does not ask me what the doctor said about me and baby´s condition when I come back from the doctor´s clinic he is careless.”* (Participant 5, gravida 3). *“When I go to visit my doctor no one from in-laws go with me due to my husband´s irresponsible behavior, he does not earn and give money to them to spend at home and on my health, so his parents dislike me. They push me to force him to do some work. I talk to him, but he does not listen to me.”*(Participant 4, gravida 4).

Lack of physical support was explained in a sense of expectations by participants´ in-laws. They said that their in-laws did not understand their feelings and pain and ask for routine work at home which was stressful for them physically as well as mentally. They added that their in-laws treated them like a maid they hired for themselves. They also said that they feel they are in the open air when they go to their parents´ homes.

*“In-laws say we are not responsible for your pregnancy, do all the work at home, we cannot give you rest if you are not able to do work so don´t bring more kids.”* (Participant 2 and 4, gravida 3, gravida 4). *“In-laws does not help me in household chores, sister-in-law eats a meal with us and does not collects utensils and does not help me in washing clothes conversely mother-in-law says to do household chores facilitates the delivery of the baby.”* (Participant 9, gravida 4). *“Our routine is that son´s wife will do all of the work at home and their own daughter will not do anything. They treat their son´s wife like a maid they hire for themselves. I feel suffocated in my husband´s home when I go to my parents´ house I feel I am in the open air.”* (Participant 10, gravida 2).

The participants experienced a lack of support from both their husbands and in-laws. In-laws did not support them when their husbands do not earn. They added that if their husbands´ financial condition was poor so the in-laws were also did not ask them whether they were in need for their health or they need any diet.

*“My husband plays with his phone and laptop and I try to talk to him, I try to spend time with him but he does not reply to my words, my mother-in-law says that although he is careless but it is the power of your prayers that you remain healthy before after delivery of the baby.”* (Participant 5, gravida 3). *“My in-laws have the poor financial condition and they know that their son also does not do any job, but they even do not ask me if I need something like food or medicine.”* (Participant 4, gravida 4).

Others highlighted that family marriages are not good as their aunts were so loving and caring before their marriage but when they turn into mothers in-law their behaviors got changed which was also painful. They expressed that they might not need medical care if they have stress free environment:

*“Mother In-law is my aunt; she liked me for her son but now she blames me for my bad luck. She says you have bad luck there is no money in your luck, so he cannot do any job. Money is in wife´s luck and kids are in husband´s luck.”* (Participant 4, gravida 4). *“She is my aunt; she was very loving when I was not married to her son but she is so harsh to me, my husband to see everything, but he does not say anything because he says environment gets tough if he says any single word to them on my behalf. Husband´s support makes you stress free during pregnancy and throughout in life.”* (I think the doctor is not needed if you are mentally relax at your husband´s home. Participant 10, gravida 2).

One of the participants´ views was against the views of all other participants; she told that her husband as well as in-laws was fine. Her husband was caring; he brought milk for her daily when she got pregnant. She added that whenever she felt any problem with her pregnancy her in-laws carried her to the doctor for checkup though they were financially poor. She said that she was satisfied with her married life.

*“My husband is so loving and caring; he takes care for my diet, he brings me ½ kg milk and fruits on daily basis, though my in-laws are poor financially but they bring me medicine if I need and they take me to the doctor if need checkup.”* (Participant 7, Primi gravida).

**Physical and mental strain:** one of the challenges faced by women was “physical and mental strain” which was due to fatigue and natural mood swinging and frustration, mothering and violence and abuse during pregnancy. Mood swinging and frustration was the most common cause of mental strain in participants. They highlighted that they feel mood changes when they get pregnant. Sometimes they do not want to hear and talk to anyone. They want mental peace during pregnancy, but their husbands do not understand them:

*“Woman gets frustrated during pregnancy, it is common. I also get frustrated, I want no one makes noise no one talk loudly, when children make noise I use abusive language to them because I dislike noise during this period. Everything returns to normal after delivery of the baby.”* (Participant 8, gravida 4). *“Woman gets frustrated during pregnancy, but husbands don´t care for their mood.”* (Participant 1, 2, 5, 9 and 10).

Mothering is also an issue during pregnancy. Participants expressed that they need help in handling other children because they cannot manage feeding and cleaning of their young children during pregnancy but their husbands and in-laws do not help them beyond watching everything.

*“To manage other children with a full term pregnancy is exhausting. My son wants to play all the time and I cannot walk easily with heavy abdomen but instead of helping my mother-in-law says not to bring other children if you are unable to control them.”* (Participant 2 gravida 3). *“It is very difficult to clean and feed other children with heavy abdomen but mother in-law says that she is not responsible for my children.”* (Participant 4 gravida 4).

Violence and abuse was also reported as a bad habit of the participant´s husband which was risky for both mother and child. One of the participants highlighted that her husband beat her without any specific reason and used abusive language with her and children also.

*“My husband beats me badly, sometimes bleeding starts from my nose.”* (Participant 6, gravida 3). *“My husband was good in the first year of marriage, but someone bewitched him, and he started using abusive language to me and children. We have been married for seven years.”* (Participant 6, gravida 3).

**Barriers to antenatal services:** participants discussed a few barriers to antenatal services in terms of socio-economic status of the family and inappropriate behavior of husbands and in-laws. Participants said that their husbands were not actively searching for a job so they were not able to pay for their antenatal checkups. Another said that their in-laws did not allow their husbands when they want to seek private health services as public sectors were not satisfactory.

*“I cannot afford maternal health services because my husband doesn´t do any work he stays home all the time.”* (Participant 4, 6, gravida 3, gravida 4). *“I want to go to the private setup but my elder sister-in-law does not allow my husband to take me to the private clinic and she goes to the private setup for herself when she gets pregnant.”* (Participant 10, gravida 2).

One of the participants gave her views that she was not allowed to go and seek maternal health services if her husband was not home and they have emergency, but they will not go alone to see the doctor.

*“If my husband is not home I am not allowed to leave home to seek medical care and my in-laws also do not go with me. Money is also a big problem for me to seek maternal health services.”* (Participant 2, gravida 3).

Participants talked about their in-laws authoritarian behavior. They stated that if their husbands give the whole salary to their mothers and if their husbands asked for money for antenatal checkup of their wives but their mother in-law did not allow them to spend money on their pregnancy. They added that they were in need to ask for money from their own parents instead of husbands or in laws because they were aware of pregnancy complications.

*“My husband wish to give me doctor´s fees but his mother did not allow him to spend on my pregnancy; he asks for money from his mother for everything he needs because he puts his full salary in his mother´s hands. I bring money from my parents for my checkups because I know complications may occur during pregnancy as I have experienced them in previous pregnancy.”* (Participant 1, gravida 2).

## Discussion

In the current study it was observed that good support from husbands and family members positively and poor support from them was negatively affect maternal and child health. It was suggested by the study participants that husband´s support provided emotional security, mental peace and improved physical health of the pregnant women. A study from Nigeria showed similar results that 86% of the women who were supported by their husbands showed less stress during pregnancy and they felt emotionally secure and physically healthy [17].

Another study in Brazil also showed that husbands supports and participation in women´ reproductive health produced feelings of confidence and safety in women [18]. Expectant mothers want to do rest in their last trimester but they have to do household chores without the help of any member from in-laws. The in-laws believed that routines tasks would facilitate the delivery of the baby. In-laws shared their experiences with their daughter in-laws that when they were young they would have to do their routine household chores without anyone´s help. The participants´ husbands also supported their parent's views. Findings were supported by a study in South Africa that daughters in-laws were expected to do harder and longer works in the fields and at their homes and in other days also during pregnancy [19].

In Pakistani culture, it is found that decision making whether it is financial or about the health of expected mothers is on the basis of hierarchy and age but a study in Nepal showed that financial decision making was mostly in the hands of husbands in the nuclear family system [20]. Multiple challenges faced by expecting mothers were the cause of physical and mental strain in them e.g. incomplete bed rest and household chores, inadequate food, use of the abusive language of family members and husbands, violent behavior of husbands i.e. beating and verbal abuse, mothering tasks like feeding and cleaning kids. They felt tired due to services they were providing to their in-laws and managing their kids without the help of anyone.

Mood swing was another big issue found in many pregnant women which was frustrating for them when no one cared for their mood. Literature showed multiple findings concordant to this study [21, 22]. They also expressed that fatigue during pregnancy was physical, psychological and emotional [21, 22]. Studies also showed that women relied financially and emotionally on their husbands and family. Poor financial support from a partner did not meet the adequate food requirements for pregnant women and affected their physical health during pregnancy [23]. Barriers that were found in accessing antenatal services were poor financial condition and husbands and in-law´s inappropriate behavior. Husbands and in-laws took pregnancy as a natural process and didn´t feel the need to go for antenatal checkups until unless complications occurred. Some risks were also understood by the participants due to overlooking behavior of husband and in-laws i.e. miscarriage in the first three months and development of high blood pressure during pregnancy. Participants understood that delay in seeking medical care may arise problems like anemia and fetal anomaly during pregnancy, but they were unable to seek medical care due to lack of financial support from husbands. Participants who were aware of the risks depended on their parents for money to prevent those risks but others who couldn´t depend on their parents and in-laws were also not supporting them, were more prone to develop such complications during pregnancy and at the time of delivery.

The findings were concordant with the study in Nepal in which role of mother-in-law was highlighted as the resource person of the family and husband had to ask money from his mother which was resulted in delayed seeking care [24]. Findings were also consistent with a systematic review which reviewed 131 studies and found all the characteristics discussed in the present study around the world in which 16% of the studies were from Africa, 29% were from Asia and others were from Latin America, Middle East [25].

The strength of the study was that it provided a richer insight of pregnant women regarding their husbands and in-laws´ support during pregnancy. Participants who were willing to participate enjoyed to give their insights and participated actively. Researcher did not feel bore or exhausted at any stage of the study. The topic was very interesting and researcher felt joy when conducting interviews.

The weakness of the study was that the topic of the study was sensitive. Researcher faced resistance in conducting interviews because whenever the title was explained to the participant they felt uneasy to provide information about their husbands and in-laws´ behavior. Only highly educated participants were convinced easily, rest of the participants needed much struggle to be taken into confidence for providing their private information. Six participants provided data only about health professionals and health services access and refused to give information about their in-laws so their recorded interviews were discarded due to insufficient data.

## Conclusion

Study findings showed that support of husbands and in-laws could affect maternal and child health both positively and negatively. The current study helps the nurses to explore pregnant women´s feelings regarding their husbands and in-laws and can treat the patient accordingly when they come to the antenatal clinics. By incorporating, support of husbands, in-laws and health professionals the comprehensive and effective support can provide optimum level of health to the expected mothers and their upcoming children to achieve the third goal in sustainable development goals. Study also suggests that nurse should provide culturally sensitive care to pregnant women.

### What is known about this topic

- Pregnancy complications are due to delay in seeking care;
- Mother in-law and husband are the key persons that if take care for pregnant lady could lead to a healthy pregnancy outcomes.

### What this study adds

- This study adds in the knowledge that there is a need of strong social support for women residing in joint family systems regarding their health and nutritional status;
- All pregnant women should be screened for depressive symptoms regardless of age, socio-economic status, and race or education level. Thorough history and physical exam could highlight pregnancy complications i.e. anemia, pregnancy induced hypertension etc. which may be because of delayed in seeking care;
- Other pregnancy related complications e.g. preterm delivery or low birth weight babies may also be assessed for poor support from husbands and In-laws during pregnancy. Case management approach could be used for pregnant women who are severely affected by their families´ behavior. Family centered approach should be used by health professionals.
